# Dietary Lysophosphatidylcholine Improves the Uptake of Astaxanthin and Modulates Cholesterol Transport in Pacific White Shrimp *Litopenaeus vannamei*

**DOI:** 10.3390/antiox13050505

**Published:** 2024-04-23

**Authors:** Ziling Song, Yang Liu, Huan Liu, Zhengwei Ye, Qiang Ma, Yuliang Wei, Lindong Xiao, Mengqing Liang, Houguo Xu

**Affiliations:** 1 College of Fisheries and Life Sciences, Shanghai Ocean University, 999 Huchenghuan Road, Shanghai 201306, China; 2 State Key Laboratory of Mariculture Biobreeding and Sustainable Goods, Yellow Sea Fisheries Research Institute, Chinese Academy of Fishery Sciences, 106 Nanjing Road, Qingdao 266071, China; 3 Weifang Key Laboratory of Precise Animal Nutrition, Weifang Kenon Biotechnology Co., Ltd., Weifang 261108, China

**Keywords:** carotenoid, lysophospholipid, lipid metabolism, antioxidant capacity, shrimp quality

## Abstract

Astaxanthin (AST), functioning as an efficient antioxidant and pigment, is one of the most expensive additives in shrimp feeds. How to improve the uptake efficiency of dietary astaxanthin into farmed shrimp is of significance. The present study investigated the effects of lysophosphatidylcholine (LPC), an emulsifier, on dietary astaxanthin efficiency, growth performance, body color, body composition, as well as lipid metabolism of juvenile Pacific white shrimp (average initial body weight: 2.4 g). Three diets were prepared: control group, the AST group (supplemented with 0.02% AST), and the AST + LPC group (supplemented with 0.02% AST and 0.1% LPC). Each diet was fed to triplicate tanks, and each tank was stocked with 30 shrimp. The shrimp were fed four times daily for eight weeks. The AST supplementation improved the growth of white shrimp, while LPC further promoted the final weight of shrimp, but the whole-shrimp proximate composition and fatty acid composition were only slightly affected by AST and LPC. The LPC supplementation significantly increased the astaxanthin deposition in the muscle. The LPC supplementation significantly increased the shell yellowness of both raw and cooked shrimp compared to the AST group. Moreover, the dietary LPC increased the high-density lipoprotein-cholesterol content but decreased the low-density lipoprotein-cholesterol content in the serum, indicating the possible regulation of lipid and cholesterol transport. The addition of astaxanthin significantly up-regulated the expression of *npc2* in the hepatopancreas compared to the control group, while the addition of LPC down-regulated the expression of *mttp* compared to the AST group. In conclusion, the LPC supplementation could facilitate the deposition of dietary astaxanthin into farmed shrimp and further enlarge the beneficial effects of dietary astaxanthin. LPC may also independently regulate shrimp body color and cholesterol transportation. This was the first investigation of the promoting effects of LPC on dietary astaxanthin efficiency.

## 1. Introduction

Pacific white shrimp, *Litopenaeus vannamei*, also known as white-leg shrimp, is the most important aquaculture shrimp species all over the world [1]. The body color of shrimp is a very important quality trait and, consequently, pigments such as natural or artificial carotenoids are commonly added into shrimp feeds to regulate the body color. Meanwhile, the prominent antioxidative effects of carotenoids are also considered when they are used [2,3].

Astaxanthin, as a non-vitamin A-derived carotenoid, is the most commonly used anti-oxidant and pigment in aqua-feeds. The use of astaxanthin in shrimp feeds has been found to not only promote the shrimp growth, survival, stress resistance, and pigmentation but also improve the antioxidant capacity and immune response [4,5,6]. However, to date, astaxanthin, whether natural or artificial, is still expensive and constitutes a large proportion of the feed cost [7]. How to increase the efficiency of dietary astaxanthin is of significance to the shrimp feed industry. Measures such as spraying post-pelleting has been tried to decrease the loss of astaxanthin during pelleting. 

Lysophosphatidylcholine (LPC), also known as lysolecithin, is being more and more commonly used as an emulsifier in animal feeds. Research on aquatic animals havs shown the positive effects of LPC on growth performance, lipid accumulation, and antioxidation status [8,9,10,11]. Because astaxanthin is lipid-soluble, LPC is assumed to be able to facilitate the uptake of dietary astaxanthin into aquatic animals. However, this speculation has not been validated. Also, little information has been available regarding the use of LPC in shrimp feeds. Therefore, the present study was aimed at investigating whether LPC supplementation can enhance the uptake and the subsequent biological activities of astaxanthin in shrimp feeds.

## 2. Materials and Methods

### 2.1. Experimental Diets and Feeding Trial

Three groups of experimental diets were prepared (Table 1). The control diet had a lipid content of 6.5%, which is suitable for Pacific white shrimp. The other two diets were prepared by adding 0.02% astaxanthin (AST, DSM Co., Ltd., Shanghai, China) or 0.02% AST + 0.1% LPC (Weifang Kenon Biotechnology Co., Ltd., Weifang, China) into the control diet. The selection of the AST dose was according to previous AST studies on the same shrimp species [4,12,13]. However, the selection of the LPC dose was according to a previous study of ours on turbot considering the lack of accurate information in shrimp [8]. In the diet preparation process, all the ingredients were firstly grinded and sieved through an 80-mesh sieve. Then, the dietary ingredients were thoroughly mixed. Oils were then added and thoroughly mixed into the ingredient mixture. Thereafter, water was added to make the dough. Pellets with 1.0 mm diameter were made using a single-screw pelleting machine. After that, the pellets were loaded into a tray and dried in an oven at 55 °C. The dry pellets were then packed and stored at −20 °C until use.

### 2.2. Experimental Shrimp and Feeding Management

The feeding experiment was conducted at the Langya Experimental Base of Yellow Sea Fisheries Research Institute (Qingdao, Shandong, China). In this experiment, Pacific white shrimp with an average initial body weight of 2.4 g were firstly acclimatized to the experimental environment with a commercial feed for two weeks. At the beginning of the feeding experiment, after fasting for 24 h, the experimental shrimp were randomly assigned to nine polyethylene tanks (300 L). In total, 3 replicate tanks were set up for each group of diet, and 30 shrimp were reared in each tank. During the experimental period, the shrimp were fed to apparent satiation four times (7:00, 12:00, 17:00, and 22:00) each day. During the shrimp rearing for eight weeks, the recirculating seawater system was turned on for two hours each day to remove the suspended solids. The system was kept hydrostatic at the rest time of the day, considering the big tank volume. The water was changed by 1/2 per day. The residual feeds and feces were siphoned out every day, and the tanks were brushed regularly from inside. During the feeding, the water temperature was 27~31 °C; salinity 28~30; dissolved oxygen > 8 mg/L; pH 7.6 to 7.9; total ammonia nitrogen ˂ 0.3 mg/L; non-ionic ammonia nitrogen ˂ 0.01 mg/L. All shrimp handling protocols, as well as the sampling protocols described below, in this study were reviewed and approved by the Animal Care and Use Committee (ACUC) of Yellow Sea Fisheries Research Institute, Chinese Academy of Fishery Sciences (protocol code ACUC202306125274; date of approval, 12 June 2023).

### 2.3. Sample Collection

At the end of the feeding experiment, after anaesthetized with eugenol, all shrimp in each tank were bulk weighed and counted. In addition, eight shrimp were randomly selected from each tank for tissue sampling. The hemolymph was collected from the pericardiac coelom of the shrimp using a 1 mL syringe. Anticoagulant was added at a 1:2 (*v*/*v*) ratio into a 1.5 mL centrifuge tube, which was centrifuged (4000× *g*, 10 min, 4 °C) after four hours to separate the plasma. After the blood was taken, three shrimp per tank were selected for the subsequent analysis of body color and proximate composition, and another three shrimp per tank were dissected for the collection of hepatopancreas and muscle samples. All tissue samples were snap-frozen in liquid nitrogen immediately after sampling and then brought back to the laboratory and stored in a −80 °C refrigerator.

### 2.4. Analysis of the Proximate Composition and Fatty Acid Composition

The proximate composition analysis of experimental diets and whole shrimp (three individual shrimp per tank) was conducted according to the standard methods of the Association of Official Analytical Chemists (AOAC). The moisture, crude protein, crude lipid, and ash were determined by the drying to constant weight in an oven at 105 °C, Kjeldahl method (FOSS KJELTEC 2300, Hillerod, Denmark), chloroform-methanol method [14], and incineration by muffle furnace at 550 °C, respectively. 

The fatty acid composition of the muscles was analyzed by gas chromatography (GC-2010 Pro, Shimadzu, Kyoto, Japan). The samples were first freeze-dried in a freeze-dryer (FDU-1100, Tokyo Rikakikai, Co., Ltd., Tokyo, Japan) for 48 h. After the lipid was extracted by the chloroform-methanol method, 30 μL of sample was placed into a 10 mL glass tube and 2 mL of 0.5 mol/L potassium hydroxide-methanol solution was added. The samples were placed into a water bath at 75 °C for 30 min. After cooling, 1 mL of boron trifluoride-methanol solution was added, and the samples were placed into a water bath again at 75 °C for 30 min. After 1 mL of water was added, 1 mL of n-hexane was added and vortexed. The solution was left on ice for 1 h, and then the supernatant was taken for the determination with gas chromatography, which was equipped with a fused silica capillary column (SH-RT-2560, 100 m × 0.25 mm × 0.20 μm, Shimadzu, Japan; dicyano-propyl-polysiloxane as stationary phase) and a flame ionization detector. The column temperature increase was programmed: from 150 °C up to 200 °C at 15 °C/min and then from 200 °C to 250 °C at 2°C/min. Both the injector and detector temperatures were 250 °C. Results were expressed as percentage of each fatty acid with respect to total fatty acids (TFAs). 

### 2.5. Analysis of Astaxanthin Concentration

The muscle astaxanthin concentration was determined by Qingdao Yuanxin Testing Technology Co., Ltd. (Qingdao, China). A total of 1 g of sample was weighed precisely and placed into a 50 mL round-bottomed centrifuge tube. Then, 4 mL of extraction solution was added and homogenized for 2 min. The homogenizer was rinsed with 4 mL of extraction solution. The two batches of solution were merged and vortexed. Then, ultrasonic extraction was carried out at <15 °C for 10 min, and centrifugation was carried out at 7104 r/min for 5 min to collect all the supernatant (into a 50 mL centrifuge tube). The residues were also collected. After centrifugation, 4 mL of extraction solution was added into the residue, and the above processes were repeated. The extracts were combined. The extracts were dehydrated by filtration with anhydrous sodium sulfate into a 100 mL brown rotary evaporation flask. The anhydrous sodium sulfate was washed with 15 mL of extract 2 times, and the extract was combined into the evaporation flask. The extract was concentrated under reduced pressure on a rotary evaporator at 40 ± 2 °C to be nearly dried. The extracts were further blow-dried with N_2_ and re-dissolved with 1.0 mL 0.1% BHT ethanol solution. The extract was filtered through 0.22 μm organic membrane before subjection to high-performance liquid chromatography (LC-2030 C 3 D, Shimadzu, Kyoto, Japan). 

### 2.6. Biochemical Parameters in Serum and Hepatopancreas

The levels of total cholesterol (TC), triglycerides (TG), high-density lipoprotein cholesterol (HDL-C), low-density lipoprotein cholesterol (LDL-C), malondialdehyde (MDA), hepatic lipase (HL), and lipoproteins esterase (LPL) in serum were measured with commercial kits. The TC, HL, and LPL kits were purchased from Beijing Solarbio Science & Technology Co., Ltd. (Beijing, China). The other kits were purchased from Nanjing Jiancheng Bioengineering Institute, China (Nanjing, China). Homogenates of hepatopancreas were prepared in 0.9% saline or 1:1 n-heptane:isopropanol according to the instructions and then centrifuged to separate the supernatant.

### 2.7. Analysis of Body Color

**Figure 1:**
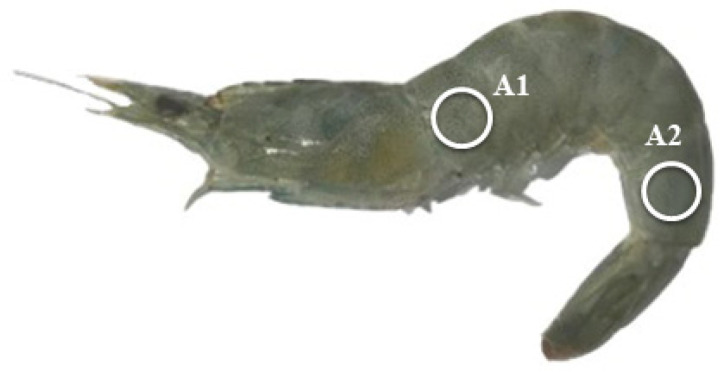
Schematic diagram of points (A1 and A2) for the shrimp body color measurement.

The raw shrimp were first wiped to clean the body surface, and then the body color was measured with a high-quality colorimeter (NR60CP+, Shenzhen ThreeNH Technology Co., Ltd., China). Two spots (Figure 1), i.e., the first one near the head (A1) and the second one near the tail (A2), were selected for the color determination. The indices L* (brightness degree), a* (redness degree), and b* (yellowness degree) were recorded. The shrimp were then placed in a self-sealing bag and boiled in 95 °C water for 3 min for the color analysis of cooked shrimp. Three shrimp per tank were used for this analysis.

### 2.8. Quantitative Real-Time Polymerase Chain Reaction (qRT-PCR)

The qRT-PCR methods (see Table 2 for primers), regents (Accurate Biotechnology and TsingKe Biological Technology, Qingdao, China), and equipment (Roche LightCycler 96, Basel, Switzerland) were the same as our previous publications [15]. The relative mRNA expression was evaluated with the 2^−ΔΔCT^ method [16]. Two reference genes, *ef-1α* and *β-actin*, which were screened according to our previous methods, were used in the calculation of mRNA expression of target genes. The geometrical mean of Ct values of these two references was used in the calculation [17]. 

### 2.9. Statistical Methods

Calculations are according to the following equations:

Weight gain g = final weight − initial weight

Weight gain % = (final weight − initial weight)/IBW × 100;

Feed conversion ratio = feed intake/weight gain;

Feed intake % = feed dry weight/[experimental days × (initial weight + final weight)/2] × 100;

Survival % = final shrimp number/initial shrimp number × 100.

All data were subjected to one-way analysis of variance (ANOVA) in SPSS 16.0 for Windows. Tukey’s multiple range test was used to detect the significant differences between the means. The significance was accepted when *p* < 0.05. The results are presented as means of triplicate tanks ± standard error.

## 3. Results

### 3.1. Growth Performances and Somatic Indices

The final weight and weight gain of the AST + LPC group was significantly (*p* < 0.05) higher than that of the control group (Table 3). There was no significant difference (*p* > 0.05) in weight gain %, feed conversation ratio, feed intake, and survival among the groups.

### 3.2. Body Proximate Composition

There was no significant (*p* > 0.05) difference in the proximate composition of whole shrimp and muscle among all groups (Table 4). The whole-shrimp crude lipid content in the AST and AST + LPC groups showed increasing trends compared to the control group.

### 3.3. Muscle Astaxanthin Concentration

**Figure 2:**
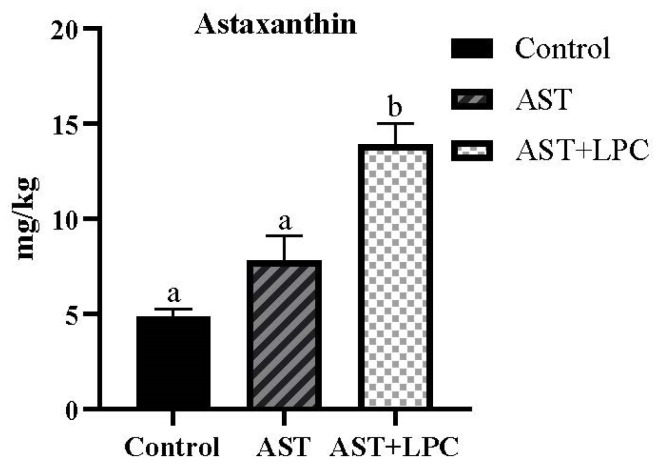
Muscle astaxanthin concentration of experimental shrimp (mean ± standard error). Data bars not sharing the same letter were significantly different (*p* < 0.05).

The astaxanthin content of the AST + LPC group was significantly (*p* < 0.05) higher than those of the other two groups (Figure 2). The astaxanthin concentration in the AST group was higher numerically compared to the control group, but there was no significant statistical difference between the two groups.

### 3.4. Body Color

**Figure 3:**
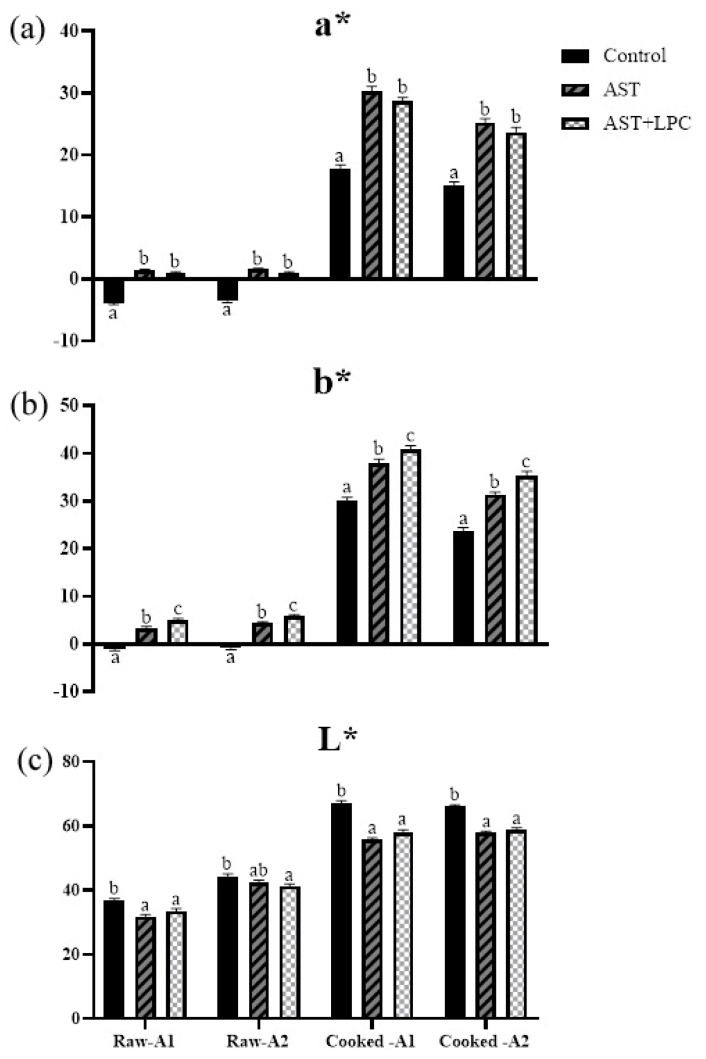
Body color parameters of experimental shrimp (mean ± standard error). For each sampling point, data bars not sharing the same letter were significantly different (*p* < 0.05). (**a**) a* value; (**b**) b* value; (**c**) L* value. The a*, b*, and L* indicate the redness (red–green), yellowness (yellow–blue), and brightness (white–black), respectively. A1 and A2 indicate the sampling points near the head and tail, respectively, as shown in Figure 1.

**Figure 4:**
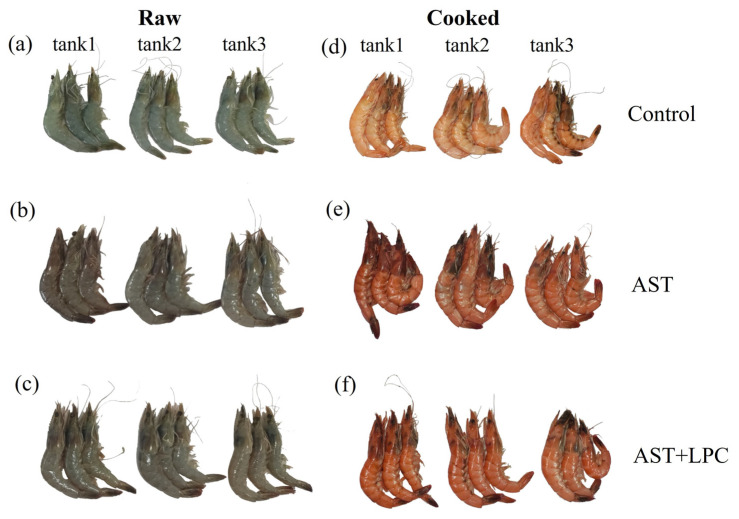
Photo pictures of raw and cooked shrimp fed experimental diets AST and LPC. (**a**) Raw—control group; (**b**) raw—AST group; (**c**) raw—AST + LPC group; (**d**) cooked—control group; (**e**) cooked—AST group; (**f**) cooked—AST + LPC group.

The L* value of shrimp body significantly (*p* < 0.05) decreased after the addition of AST (Figure 3 and Figure 4). The a* value was significantly (*p* < 0.05) increased by the addition of AST, but the addition of LPC slightly decreased the a* value compared to the AST group. The b* values significantly (*p* < 0.05) ranked as follows: AST + LPC > AST > control.

### 3.5. Fatty Acid Profiles of the Hepatopancreas

The contents of 14:1n-5, 16:1n-7, 18:1n-9, and mono-unsaturated fatty acids (MUFAs) were significantly (*p* < 0.05) lower in the AST group than in the other two groups, and there was no significant (*p* > 0.05) difference between the control and LPC + AST groups (Table 5). The contents of 18:2n-6 and n-6 polyunsaturated fatty acid (PUFA) were significantly (*p* < 0.05) higher in the LPC + AST group than those in the control group, and there was no significant (*p* > 0.05) difference between the control and AST groups. The n-3 PUFA content and n-3/n-6 ratio were lower in the LPC + AST group than those in the other two groups, and there was no significant (*p* > 0.05) difference between the control and AST groups.

### 3.6. Biochemical Parameters in Serum and Hepatopancreas

**Figure 5:**
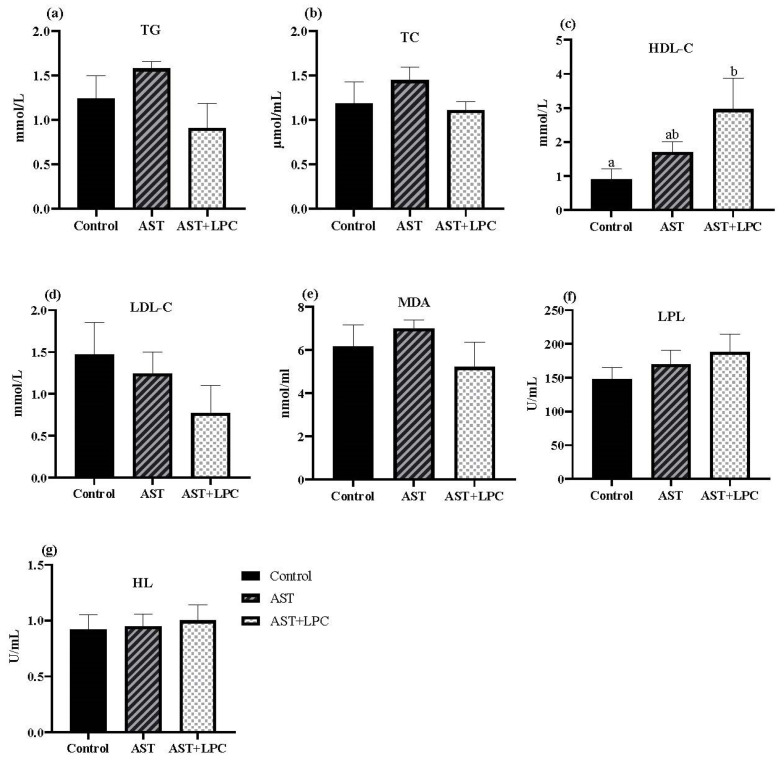
Plasma biochemical parameters of experimental shrimp (mean ± standard error). (**a**) Triglycerides (TG); (**b**) total cholesterol (TC); (**c**) high-density lipoprotein cholesterol (HDL-C); (**d**) low-density lipoprotein cholesterol (LDL-C); (**e**) malondialdehyde (MDA); (**f**) lipoproteins lipase (LPL); (**g**); and hepatic lipase (HL). Data bars not sharing the same superscript letter were significantly different (*p* < 0.05).

There was no significant (*p* > 0.05) difference in serum TG, TC, LDL-C, MDA, LPL, and HL contents among all groups (Figure 5). However, the TG, TC, and MDA contents tended to be lower in the AST + LPC group compared to the AST group. The HDL-C content in the AST + LPC group was significantly (*p* < 0.05) higher than that in the control group. The LPL content showed a similar trend, but no significant difference was observed among groups. 

**Figure 6:**
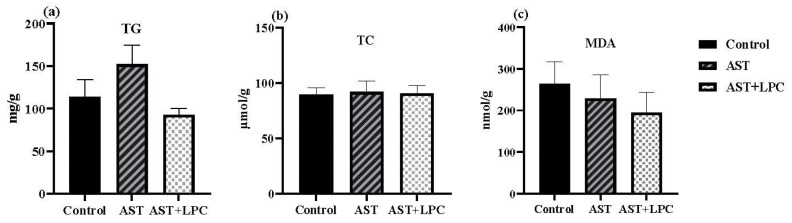
Hepatopancreas biochemical parameters of experimental shrimp (mean ± standard error). (**a**) Triglycerides (TG); (**b**) total cholesterol (TC); (**c**) malondialdehyde (MDA).

There was no significant (*p* > 0.05) difference in hepatopancreas TG, TC, and MDA contents among the groups (Figure 6). The lowest TG content was observed in the AST group, and the lowest MDA content was observed in the AST + LPC group.

### 3.7. mRNA Expression of Genes Related to Lipid and Cholesterol Transport

**Figure 7:**
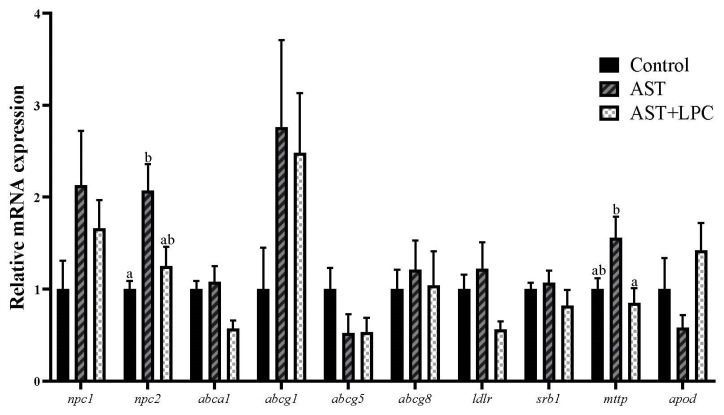
Relative mRNA expression of genes related to lipid and cholesterol transport in the hepatopancreas of experimental shrimp (mean ± standard error). Data bars not sharing a same lowercase letter were significantly different (*p* < 0.05).

In the hepatopancreas of shrimp (Figure 7), the experimental diets significantly (*p* < 0.05) regulated the expression of only *npc2* and *mttp*, both of which had the highest expression levels in the AST group. Most other genes, except *abcg5* and *apod*, showed similar trends to *npc2* and *mttp* in response to diets, although no significant (*p* > 0.05) difference was observed among groups.

## 4. Discussion

The beneficial effects of astaxanthin have been widely demonstrated in aquatic animals in terms of growth, survival, immunity, and anti-oxidation capacity [18,19,20,21,22,23]. These beneficial effects were also observed in this study, which clearly showed the growth-stimulating activity of astaxanthin (0.02%) in Pacific white shrimp. This result was consistent with the results of other studies on white shrimp [4,24,25]. Considering that the anti-oxidation capacity of astaxanthin has been a widely demonstrated and even undeniable fact, this anti-oxidation capacity was not further investigated and discussed in this study. We preferred to rather focus on effects of LPC on AST efficacy. In the present study, the growth was further improved by the addition of AST + LPC. This improvement could be, at least partly, attributed to the modulation of anti-oxidative status. Previous studies on crayfish *Procambarus clarkii* have evidenced that LPC could improve shrimp growth independently, probably by improving lipids absorption [9]. Unfortunately, in this study, there was no independent LPC treatment group, since the main purpose of this study was to evaluate the AST-stimulating effects of LPC. There was no significant difference in survival among experimental groups. The survival in all groups was higher than 95%, also indicating the accuracy of the feeding trial. Nevertheless, the improvement of survival by LPC has been observed in other fish and shrimp studies [4,8,9]. 

Regarding the body proximate composition, there were no significant difference in content of moisture, crude protein, and crude lipid of whole shrimp and muscle among groups. In studies on rainbow trout *Oncorhynchus mykiss* [10,26], channel catfish *Ictalurus punctatus* [11], and tiger puffer *Takifugu rubripes* [27], it has been observed that astaxanthin and LPC could promote lipid digestion and absorption and may thus increase the body lipid content. However, in this study, only an increasing trend in lipid content was observed from the control group to the AST + LPC group, but no significant differences were observed. The large intra-group data deviations could mask the significant differences between groups.

The most important aim of this study was to investigate the AST deposition-stimulating effects of LPC, as well as the subsequent effects on shrimp body color. These effects were indeed observed in this study, in particular regarding the muscle astaxanthin content. The elevation of muscle astaxanthin content by LPC was expectable considering that astaxanthin is lipid-soluble and LPC is a high-quality lipid emulsifier. However, this was the first evidence of this stimulating effect, and the stimulating efficiency was quite high. The muscle astaxanthin content in the AST + LPC group was even two times higher than that of the AST group. Considering that the astaxanthin, whether natural or artificial, is still expensive, sometime constituting 1/4–1/3 of the salmon feed cost, this result is of significance to the aquafeed industry. 

Subsequently, this AST deposition-stimulating effect of LPC was reflected by the shrimp body color. Although the difference in body color between the AST and AST + LPC groups can barely be observed by naked eyes, this difference can be clearly detectable by machine [28]. In this study, for both raw and cooked shrimp, the dietary supplementation of astaxanthin significantly increased the redness but decreased the brightness. This result was similar to what was observed in other crustaceans and fish species [29,30,31,32,33,34]. More importantly, compared to the AST group, the supplementation of LPC significantly further increased the yellowness but did not affect the redness and brightness. This was quite a novel finding. 

Regarding the muscle fatty acid composition, the supplementation of astaxanthin and LPC resulted in only minor changes. The increase of 18:2n-6 and n-6 PUFA contents by AST + LPC was similar to what was observed in stellate sturgeon *Acipenser stellatus* [35]. This result was probably due to the fact that the LPC used in this study was derived from soybean, which is rich in 18:2n-6 and n-6 PUFA. Some other minor changes such as those in 16:1n-7 and 18:1n-9 were difficult to explain based on the current information. A recent study on turbot also found that LPC led to minor changes in body fatty acid composition [8].

In both terrestrial animals and aquatic animals, it has been found that the effects of LPC, although mainly acting as an emulsifier, were not restricted to lipid digestion and absorption [36,37]. In fish and shrimp such as turbot *Scophthalmus maximus* [8,38], rainbow trout [10,26], channel catfish [11], and tiger shrimp *Penaeus monodon* [39], LPC has also been used to overcome or mitigate the lipid metabolism disorders caused by high dietary lipid levels or fish oil replacement. In previous studies, it was observed that dietary supplementation of LPC reduced the systemic lipid levels or serum levels of TC and TG [8,9,11,26], although some other studies did not observe these effects [39,40]. In this study, dietary AST and LPC had marginal effects on serum TG and TC levels. Species, feeding duration, and dietary formulation may largely influence the lipid-regulating effects of LPC. 

However, it was clear that the astaxanthin supplementation increased the serum HDL-C concentration and decreased the LDL-C content. Moreover, LPC amplified this effect. LPL and HL also showed similar trends. Lipoprotein lipase (LPL) and hepatic lipase (HL), synthesized by liver cells, are key enzymes that regulate lipid metabolism and catalyze the hydrolysis of triglycerides. Increased serum levels or activities of LPL and HL can lead to increased lipid clearance, resulting in reduced TG and metabolic disorder mitigation [41]. Considering the roles of the parameters mentioned above in lipid and cholesterol transport, it was speculated that LPC may be involved in the transport of lipid and cholesterol. During blood circulation, LDL carries cholesterol from the liver to peripheral tissues for utilization, while HDL carries cholesterol from peripheral tissues to the liver for cholesterol removal [42,43]. Therefore, the LDL-C/HDL-C ratio is often used as a marker of cholesterol transport and an indicator of cholesterol accumulation in peripheral vasculature [44]. Results of this study suggested that the AST and LPC facilitated reverse cholesterol transport, i.e., the transport of cholesterol from extrahepatic tissue towards the liver for eventual excretion [45,46,47]. In particular, LPC facilitated the effects of AST in this process. 

Nevertheless, from the aspect of gene expression, only the mRNA expressions of *npc2* and *mttp* were significantly affected by the diets. Npc1 and Npc2 are key proteins involved in the intracellular lipid transport. Cholesterol is firstly bound to Npc2 and then transferred to Npc1, thus increasing the rate of cholesterol transport from Npc1 to intestinal epithelial cells [48]. Mttp is not a protein specifically associated with cholesterol metabolism but a protein widely involved in the formation and secretion of ApoB-rich lipoproteins, especially in the pathways associated with chylomicrons and VLDL [49]. In general, the down-regulation of *npc2* and *mttp* mRNA expression by LPC compared to the AST group may be related to the inhibition of cholesterol absorption in the intestine. 

In addition to cholesterol metabolism, the MDA content in the hepatopancreas tended to be reduced by both AST and AST + LPC. MDA is a main end-product of lipid peroxidation [50]. This result was not surprising considering the facts that astaxanthin is a strong antioxidant and that LPC promoted astaxanthin deposition. The anti-oxidative capacity of astaxanthin has been widely observed in other aquatic species [30,31,32,33,51,52] and thus was not discussed in detail in this study.

## 5. Conclusions

In conclusion, the dietary supplementation of lysophosphatidylcholine (LPC) improved the astaxanthin deposition in Pacific white shrimp and amplified its beneficial effects on growth performance and body color. The dietary LPC significantly increased the shell yellowness of both raw and cooked shrimp. LPC may also regulate the lipid and cholesterol transport in white shrimp. This was the first investigation of the promoting effects of LPC on dietary astaxanthin efficiency.

## Figures and Tables

**Table 1:** The formulation and proximate composition of the experimental diets used in this study (% dry matter except moisture).

Ingredient	Control	AST	AST + LPC
Fish meal	20	20	20
Soybean meal	30	30	30
Peanut meal	14	14	14
Poultry by-product meal	4	4	4
Wheat meal	21	21	21
Mineral premix ^a^	0.5	0.5	0.5
Vitamin premix ^a^	1	1	1
Monocalcium phosphate	1	1	1
Vitamin C	0.2	0.2	0.2
Choline chloride	0.2	0.2	0.2
Ethoxyquin	0.02	0.02	0.02
Mold inhibitor	0.1	0.1	0.1
Betaine	0.3	0.3	0.3
Soya lecithin	1.5	1.5	1.5
Soybean oil	3	3	3
CarophyllPink ^b^	0	0.2	0.2
Lysophosphatidylcholine ^c^	0	0	0.1
Y_2_O_3_	0.1	0.1	0.1
Bentonite	1.08	0.88	0.78
Alginate	2	2	2
Proximate composition			
Crude protein	43.9	44.0	44.5
Crude lipid	6.5	6.4	6.7
Ash	10.3	10.5	10.2
Moisture	8.3	8.1	8.5
Gross energy (MJ/kg)	17.8	17.8	17.7

^a^ Vitamin premix and mineral premix, designed for marine fish, were purchased from Qingdao Master Biotech Co., Ltd., Qingdao, China. Generally, the vitamin premix contained retinyl acetate, vitamin D_3_, DL-α-tocopherol acetate, menadione nicotinamide bisulfite, thiamine, riboflavin, vitamin B6, cyanocobalamin, D-calcium pantothenate, niacinamide, folic acid, D-biotin, L-ascorbate-2-phosphate, inositol, betaine hydrochloride, yeast hydrolysate, and rice hull powder; the mineral premix contained ferrous sulfate, zinc sulphate, manganese sulphate, cupric sulfate, cobaltous chloride, sodium selenite, calcium iodate, and zeolite powder. ^b^ Astaxanthin was added as Carophyllpink of DSM Co., Ltd. (Shanghai, China), which contained 10% astaxanthin. ^c^ The phosphatidylcholine supplied by Weifang Kenon Biological Technology Co., Ltd. (Weifang, China) had a purity of 98%, and the available LPC concentration was 5%.

**Table 2:** Sequences of the PCR primers used in this work.

Primer	Forward Primer (5′−3′)	GenBank Reference	Product Length (bp)
*npc1*-F	CGAAGGGGAAAAGCCAGAGT	XM_027363410.1	87
*npc1*-R	TTGAGGAGGAAGGGAGCGTA		
*npc2*-F	CGCAGTATCGGCAGTCAAGA	XM_027358057.1	149
*npc2*-R	GTGGTGTGAAAGGCAACGAC		
*abca1*-F	ACCTGAAGGCAGGACGAAAG	XM_027375794.1	73
*abca1*-R	GGCATACTCGCGCTATCTGT		
*abcg1*-F	TCCCGGAAGGCAGGAATAGA	XM_027356334.1	121
*abcg1*-R	TTCATCAGCGTCGACTTCCC		
*abcg5*-F	TCCGTTTGCCCCGATAACAA	XM_027382028.1	74
*abcg5*-R	TAGCGCTCGAGCAGGTAGTA		
*abcg8*-F	CCAACGATTCCGAAGGGTCT	XM_027368984.1	93
*abcg8*-R	CGTTGAGGATGAAGTCCCCC		
*ldlr*-F	CGTCACATGCCCAGCCATAA	XM_027363319.1	86
*ldlr*-R	GATCCGTCATGGCACTCGAA		
*srb1*-F	GTTCGACATCTACCCGGACC	XM_027352601.1	132
*srb1*-R	AACCAGAAGATGGGCAGGAC		
*mttp*-F	GCTGCTAAGGAAAGTGCGTG	XM_027380336.1	215
*mttp*-R	AAGGATGCGTCGCTAAGGAG		
*apod*-F	CAACGCGGTAACAGGGAAAG	XM_027368898.1	85
*apod*-R	GACAACCAGCTTGGCTTCAC		
*ef-1α*-F	GTATTGGAACAGTGCCCGTG	GU136229.1	143
*ef-1α*-R	ACCAGGGACAGCCTCAGTAAG		
*β-actin*-F	CGAGGTATCCTCACCCTGAA	AF300705.2	176
*β-actin*-R	GTCATCTTCTCGCGGTTAGC		

*npc*: NPC intracellular cholesterol transporter; *abca*: ATP-binding cassette sub-family A; *abcg*: ATP-binding cassette sub-family G; *ldlr*: low-density lipoprotein receptor; *srb1*: scavenger receptor class B member 1; *mttp*: microsomal triglyceride transfer protein large subunit; *apod*: apolipoprotein D.

**Table 3:** Growth performance of experimental shrimp (mean ± standard error).

Parameter	Control	AST	AST + LPC	*p* Value
Initial weight g	2.41 ± 0.09	2.34 ± 0.04	2.58 ± 0.05	0.162
Final weight g	10.35 ± 0.03 ^a^	10.96 ± 0.25 ^ab^	11.45 ± 0.34 ^b^	0.046
Weight gain g	7.94 ± 0.07 ^a^	8.62 ± 0.21 ^ab^	8.87 ± 0.29 ^b^	0.041
Weight gain %	330.33 ± 14.31	367.67 ± 3.28	344.00 ± 5.00	0.229
Feed conversion ratio	1.35 ± 0.06	1.24 ± 0.05	1.24 ± 0.04	0.573
Feed intake %	2.93 ± 0.14	2.83 ± 0.10	2.82 ± 0.01	0.792
Survival %	95.33 ± 2.33	98.00 ± 1.00	98.50 ± 1.50	0.460

Data in the same row not sharing a superscript letter were significantly (*p* < 0.05) different.

**Table 4:** Body proximate composition of experimental shrimp (% wet weight, mean ± standard error).

Parameter	Control	AST	AST + LPC	*p* Value
Whole shrimp				
Moisture	77.20 ± 0.61	76.71 ± 0.68	76.76 ± 1.03	0.894
Crude protein	17.59 ± 0.47	17.72 ± 0.42	17.83 ± 0.58	0.943
Crude lipid	0.68 ± 0.07	1.32 ± 0.26	1.38 ± 0.38	0.583
Ash	3.10 ± 0.12	2.90 ± 0.10	2.90 ± 0.20	0.582
Muscle				
Moisture	74.74 ± 0.40	74.36 ± 1.11	74.33 ± 0.32	0.902
Crude protein	22.65 ± 0.29	22.80 ± 82	22.58 ± 0.33	0.959
Crude lipid	1.15 ± 0.08	1.13 ± 0.07	1.17 ± 0.02	0.873

**Table 5:** Muscle fatty acid composition of experimental shrimp (%TFA, mean ± standard error).

Fatty Acid	Control	AST	LPC + AST	*p* Value
14:0	0.33 ± 0.04	0.21 ± 0.01	0.33 ± 0.04	0.093
16:0	19.54 ± 0.29	19.49 ± 0.18	20.07 ± 1.00	0.764
18:0	9.21 ± 0.09	9.96 ± 0.24	9.32 ± 0.26	0.098
20:0	0.19 ± 0.00	0.19 ± 0.00	0.19 ± 0.01	0.527
SFA	29.26 ± 0.28	29.85 ± 0.06	29.91 ± 0.86	0.648
14:1n-5	0.20 ± 0.02 ^b^	0.14 ± 0.01 ^a^	0.19 ± 0.02 ^ab^	0.035
16:1n-7	1.10 ± 0.08 ^b^	0.76 ± 0.04 ^a^	1.07 ± 0.06 ^b^	0.013
18:1n-9	13.44 ± 0.13 ^ab^	12.83 ± 0.15 ^a^	13.90 ± 0.21 ^b^	0.011
22:1n-9	0.16 ± 0.00	0.17 ± 0.01	0.18 ± 0.01	0.530
MUFA	14.90 ± 0.11 ^b^	13.90 ± 0.19 ^a^	15.35 ± 0.25 ^b^	0.005
8:2n-6	18.58 ± 0.32 ^a^	18.88 ± 0.42 ^ab^	19.99 ± 0.04 ^b^	0.038
20:2n-6	1.54 ± 0.03	1.72 ± 0.08	1.59 ± 0.08	0.242
n-6 PUFA	21.82 ± 0.31 ^a^	22.45 ± 0.41 ^ab^	23.20 ± 0.17 ^b^	0.055
18:3n-3	1.05 ± 0.03	0.99 ± 0.06	1.10 ± 0.04	0.292
20:3n-3	1.69 ± 0.03	1.84 ± 0.08	1.62 ± 0.13	0.295
20:5n-3	9.39 ± 0.15	9.75 ± 0.22	8.61 ± 0.37	0.057
22:5n-3	0.84 ± 0.05	0.77 ± 0.04	0.83 ± 0.03	0.456
22:6n-3	10.41 ± 0.19	10.14 ± 0.24	9.58 ± 0.23	0.092
n-3 PUFA	23.38 ± 0.07 ^ab^	23.48 ± 0.16 ^b^	21.73 ± 0.64 ^a^	0.031
n-3/n-6	0.99 ± 0.01 ^b^	0.97 ± 0.02 ^b^	0.87 ± 0.02 ^a^	0.004

Data in the same row not sharing a superscript letter were significantly (*p* < 0.05) different. SFA: saturated fatty acid; MUFA: mono-unsaturated fatty acids; PUFA: polyunsaturated fatty acid.

## Data Availability

The raw data supporting the conclusions of this article will be made available by the authors on request.

## References

[1] Chen Y.K., Mitra A., Rahimnejad S., Chi S.Y., Kumar V., Tan B.P., Niu J., Xie S.W. (2023). Retrospect of fish meal substitution in Pacific white shrimp (*Litopenaeus vannamei*) feed: Alternatives, limitations and future prospects. Rev. Aquac..

[2] Nishida Y., Berg P.C., Shakersain B., Hecht K., Takikawa A., Tao R., Kakuta Y., Uragami C., Hashimoto H., Misawa N. (2023). Astaxanthin: Past, present, and future. Mar. Drugs.

[3] Matsuno T. (2001). Aquatic animal carotenoids. Fish. Sci..

[4] Niu J., Tian L.X., Liu Y.J., Yang H.J., Ye C.X., Gao W., Mai K.S. (2009). Effect of dietary astaxanthin on growth, survival, and stress tolerance of postlarval shrimp, *Litopenaeus vannamei*. J. World Aquac. Soc..

[5] Zhang J., Liu Y.J., Tian L.X., Yang H.J., Liang G.Y., Yue Y.R., Xu D.H. (2013). Effects of dietary astaxanthin on growth, antioxidant capacity and gene expression in Pacific white shrimp *Litopenaeus vannamei*. Aquac. Nutr..

[6] Wang W.L., Ishikawa M., Koshio S., Yokoyama S., Dawood M.A.O., Hossain M.S., Zaineldin A.I. (2019). Interactive effects of dietary astaxanthin and cholesterol on the growth, pigmentation, fatty acid analysis, immune response and stress resistance of kuruma shrimp (*Marsupenaeus japonicus*). Aquac. Nutr..

[7] Nguyen K.D. (2013). Astaxanthin: A Comparative Case of Synthetic vs. Natural Production.

[8] Xu H.G., Luo X., Bi Q.Z., Wang Z.D., Meng X.X., Liu J.S., Duan M., Wei Y.L., Liang M.Q. (2022). Effects of dietary lysophosphatidylcholine on growth performance and lipid metabolism of juvenile turbot. Aquac. Nutr..

[9] Cai M.L. (2021). Effects of Dietary Lysolecithin on Growth, Lipid Metabolism and Muscle Quality of Red Swamp Crayfish (*Procambarus clarkii*). Master’s Thesis.

[10] Taghavizadeh M., Shekarabi H.P.S., Mehrgan S.M., Islami H.R. (2020). Efficacy of dietary lysophospholipids (Lipidol™) on growth performance, serum immuno-biochemical parameters, and the expression of immune and antioxidant-related genes in rainbow trout (*Oncorhynchus mykiss*). Aquaculture.

[11] Liu G.X., Ma S.L., Chen F.Y., Zhang W.B., Mai K.S. (2020). Effects of dietary lysolecithin on growth performance, feed utilization, intestinal morphology and metabolic responses of channel catfish (*Ictalurus punctatus*). Aquac. Nutr..

[12] Eldessouki E.A.A., Diab A.M., Selema T.A.M.A., Sabry N.M., Abotaleb M.M., Khalil R.H., Abdel T.M. (2022). Dietary astaxanthin modulated the performance, gastrointestinal histology, and antioxidant and immune responses and enhanced the resistance of *Litopenaeus vannamei* against *Vibrio harveyi* infection. Aquac. Int..

[13] Samia F., Wang W.L., Zhou Y., Xue Y.C., Yi G.F., Wu M.Q., Huang X.X. (2022). Can dietary β-carotene supplementation provide an alternative to astaxanthin on the performance of growth, pigmentation, biochemical, and immuno-physiological parameters of *Litopenaeus vannamei*?. Aquac. Rep..

[14] Folch J., Lee M., Sloane-Stanley G.H. (1957). A simple method for the isolation and purification of total lipids from animal tissues. J. Biol. Chem..

[15] Meng X.X., Bi Q.Z., Cao L., Ma Q., Wei Y.L., Duan M., Liang M.Q., Xu H.G. (2022). Evaluation of necessity of cholesterol supplementation in diets of two marine teleosts, turbot (*Scophthalmus maximus*) and tiger puffer (*Takifugu rubripes*): Effects on growth and lipid metabolism. Aquac. Nutr..

[16] Livak K.J., Schmittgen T.D. (2001). Analysis of relative gene expression data using real–time quantitative PCR and the 2^−ΔΔCT^ method. Methods.

[17] Liao Z.B., Sun Z.Y., Bi Q.Z., Gong Q.L., Sun B., Wei Y.L., Liang M.Q., Xu H.G. (2021). Screening of reference genes in tiger puffer (*Takifugu rubripes*) across tissues and under different nutritional conditions. Fish. Physiol. Biochem..

[18] Liu L., Li J., Cai X.N., Ai Y., Long H., Ren W., Huang A.Y., Zhang X., Xie Z.Y. (2022). Dietary supplementation of astaxanthin is superior to its combination with Lactococcus lactis in improving the growth performance, antioxidant capacity, immunity and disease resistance of white shrimp (*Litopenaeus vannamei*). Aquac. Rep..

[19] Keng C.L., Yusoff M.F., Shariff M., Kamarudin M.S., Nagao N. (2019). Dietary supplementation of astaxanthin enhances hemato-biochemistry and innate immunity of Asian seabass, *Lates calcarifer* (Bloch, 1790). Aquaculture.

[20] Wang W.L., Ishikawam M., Koshio S., Yokoyama S., Dawood M.A., Zhang Y.K. (2018). Effects of dietary astaxanthin supplementation on survival, growth and stress resistance in larval and post-larval kuruma shrimp, *Marsupenaeus japonicus*. Aquac. Res..

[21] Song X.L., Wang L., Li X.Q., Chen Z.Z., Liang G.Y., Leng X.J. (2017). Dietary astaxanthin improved the body pigmentation and antioxidant function, but not the growth of discus fish (*Symphysodon* spp.). Aquac. Res..

[22] Xie S., Yin P., Tian L., Yu Y., Liu Y., Niu J. (2020). Dietary supplementation of astaxanthin improved the growth performance, antioxidant ability and immune response of juvenile largemouth bass (*Micropterus salmoides*) fed high-fat diet. Mar. Drugs.

[23] Xie J.J., Chen X., Liu Y.J., Tian L.X., Xie S.W., Niu J. (2017). Effects of dietary astaxanthin on growth performance, hepatic antioxidative activity, hsp70, and HIF-1α gene expression of juvenile golden pompano (*Trachinotus ovatus*). Isr. J. Aquac.-Bamidgeh.

[24] Fang H.H., He X.S., Zeng H.L., Liu Y.J., Tian L.X., Niu J. (2021). Replacement of astaxanthin with lutein in diets of juvenile *Litopenaeus vannamei*: Effects on growth performance, antioxidant capacity, and immune response. Front. Mar. Sci..

[25] Tageldein A.M., Mohamed A., Eman M.A., Ahmed S.A., Mahmoud S.K., Mohamed A.E., Zaki Z.S. (2022). Growth performance, immune-related and antioxidant genes expression, and gut bacterial abundance of pacific white leg shrimp, *Litopenaeus vannamei*, dietary supplemented with natural astaxanthin. Front. Physiol..

[26] Batoul A., Keramat A.A., Hosein O., Mohamad K., Soleiman M. (2021). Effects of lysophospholipid on rainbow trout (*Oncorhynchus mykiss*) growth, biochemical indices, nutrient digestibility and liver histomorphometry when fed fat powder diet. Aquac. Nutr..

[27] Liao Z.B., Xu H.G., Wei Y.L., Zhang Q.G., Liang M.Q. (2018). Dietary astaxanthin differentially affected the lipid accumulation in the liver and muscle of the marine teleost, tiger puffer *Takifugu rubripes*. Aquac. Res..

[28] Smith B.E., Hardy R.W., Torrissen O.J. (1992). Synthetic astaxanthin deposition in pan-size coho salmon (*Oncorhynchus kisutch*). Aquaculture.

[29] Supamattaya K., Kiriratnikom S., Boonyaratpalin M., Borowitzka L. (2005). Effect of a dunaliella extract on growth performance, health condition, immune response and disease resistance in black tiger shrimp (*Penaeus monodon*). Aquaculture.

[30] Zhang J.J., Li X.Q., Leng X.J., Zhang C.L., Han Z.Y., Zhang F.G. (2013). Effects of dietary astaxanthins on pigmentation of flesh and tissue antioxidation of rainbow trout (*Oncorhynchus mykiss*). Aquac. Int..

[31] Chen Q., Huang S., Dai J.Y., Wang C.C., Chen S.M., Qian Y.X., Han T. (2023). Effects of synthetic astaxanthin on the growth performance, pigmentation, antioxidant capacity, and immune response in black tiger prawn (*Penaeus monodon*). Aquac. Nutr..

[32] Huang S., Chen Q., Zhang M.M., Chen S.M., Dai J.Y., Qian Y.X., Gong Y.Y., Han T. (2023). Synthetic astaxanthin has better effects than natural astaxanthins on growth performance, body color and n-3 PUFA deposition in black tiger prawn (*Penaeus monodon*). Aquac. Rep..

[33] Zhao X.P., Wang G.P., Liu X.G., Guo D.L., Chen X.L., Liu S., Li G.F. (2022). Dietary supplementation of astaxanthin increased growth, colouration, the capacity of hypoxia and ammonia tolerance of Pacific white shrimp (*Litopenaeus vannamei*). Aquac. Rep..

[34] Lin Y.J., Chang J.J., Huang H.T., Lee C.P., Hu Y.F., Wu M.L., Huang C.Y., Nan F.H. (2023). Improving red-colorperformance, immune response and resistance to *Vibrio parahaemolyticus* on white shrimp *Penaeus vannamei* by an engineered astaxanthin yeast. Sci. Rep..

[35] Fatemeh J., Naser A., Farzaneh N., Enric G., Mansour T.M. (2024). Supplementing lysolecithin in corn-oil based diet enhanced growth and improved body biochemical composition in juvenile stellate sturgeon (*Acipenser stellatus*). Anim. Feed. Sci. Technol..

[36] Juntanapum W., Bunchasak C., Poeikhampha T., Rakangthong C., Poungpong K. (2020). Effects of supplementation of lysophosphatidylcholine (LPC) to lying hens on production performance, fat digestibility, blood lipid profile, and gene expression related to nutrients transport in small intestine. J. Anim. Feed Sci..

[37] Nutautaitė M., Racevičiūtė S.A., Andalibizadeh L., Šašytė V., Bliznikas S., Pockevičius A., Vilienė V. (2021). Improving broiler chickens’ health by using lecithin and lysophosphatidylcholine emulsifiers: A comparative analysis of physiological indicators. Iran. J. Vet. Res..

[38] Li S.H., Luo X., Liao Z.B., Liang M.Q., Xu H.G., Mai K.S., Zhang Y.J. (2022). Effects of lysophosphatidylcholine on intestinal health of turbot fed high-lipid diets. Nutrients.

[39] Imran H.K., Syama J.D., Kondusamy A., Purdhvi E.M., Rajabdeen J., Vanjiappan R. (2018). Enhancing the dietary value of palm oil in the presence of lysolecithin in tiger shrimp, *Penaeus monodon*. Aquac. Int..

[40] Li H.T., Tian L.X., Wang Y.D., Hu Y.H. (2010). Effects of lysolecithin on growth performance, body composition and hematological indices of hybrid tilapia (*Oreochromis aureus* ♂×*Oreochromis niloticus* ♀). J. Dalian Fish. Univ..

[41] Wang M., Wu X.H., Li Z. (2014). Leptin accelerates lipid metabolism by increasing lipoprotein lipase and hepatic lipase expression in insulin-resistant liver cell model. J. Third Mil. Med. Univ..

[42] Chen J.Y., Chen J.C., Wu J.L. (2003). Molecular cloning and functional analysis of zebrafish high-density lipoprotein-binding protein Comp. Comp. Biochem. Physiol. Part B Biochem. Mol. Biol..

[43] Deng J., Mai K., Ai Q., Zhang W., Wang X., Tan B., Xu W., Liu Z., Ma H. (2010). Interactive effects of dietary cholesterol and protein sources on growth performance and cholesterol metabolism of Japanese flounder (*Paralichthys olivaceus*). Aquac. Nutr..

[44] Yun B., Mai K.S., Zhang W.B., Xu W. (2011). Effects of dietary cholesterol on growth performance, feed intake and cholesterol metabolism in juvenile turbot (*Scophthalmus maximus* L.) fed high plant protein diets. Aquaculture.

[45] Mardones P., Quiñones V., Amigo L., Moreno M., Miquel J.F., Schwarz M., Miettinen H.E., Trigatti B., Krieger M., VanPatten S. (2001). Hepatic cholesterol and bile acid metabolism and intestinal cholesterol absorption in scavenger receptor class B type I-deficient mice. J. Lipid Res..

[46] Stanley S.L., Stephen G.W. (2015). Implications of reverse cholesterol transport: Recent studies. Clin. Chim. Acta.

[47] Brufau G., Groen A.K., Kuipers F. (2011). Reverse cholesterol transport revisited: Contribution of biliary versus intestinal cholesterol excretion. Arterioscler. Thromb. Vasc. Biol..

[48] Wang L.M., Motamed M., Infante E.R., Abi-Mosleh L., Kwon H.J., Brown M.S., Goldstein J.L. (2010). Identification of Surface Residues on Niemann-Pick C2 Essential for Hydrophobic Handoff of Cholesterol to NPC1 in Lysosomes. Cell Metab..

[49] Wei F., Chen Q.C., Cui K., Chen Q., Li X.S., Xu N., Mai K.S., Ai Q.H. (2021). Lipid overload impairs hepatic VLDL secretion via oxidative stress-mediated PKCδ-HNF4α-MTP pathway in large yellow croaker (*Larimichthys crocea*). Free Radic. Biol. Med..

[50] Zhang D.G., Zhao T., Hogstrand C., Ye H.M., Xu X.J., Luo Z. (2021). Oxidized fish oils increased lipid deposition via oxidative stress-mediated mitochondrial dysfunction and the CREB1-Bcl2-Beclin1 pathway in the liver tissues and hepatocytes of yellow catfish. Food Chem..

[51] Brambilla F., Forchino A., Antonini M., Rimoldi S., Terova G., Saroglia M. (2009). Effect of dietary astaxanthin sources supplementation on muscle pigmentation and lipid peroxidation in rainbow trout (*Oncorhynchus mykiss*). Ital. J. Anim. Sci..

[52] Xu W.X., Liu Y.T., Huang W.X., Yao C.W., Yin Z.Y., Mai K.S., Ai Q.H. (2022). Effects of dietary supplementation of astaxanthin (Ast) on growth performance, activities of digestive enzymes, antioxidant capacity and lipid metabolism of large yellow croaker (*Larimichthys crocea*) larvae. Aquac. Res..

